# Sleep duration modifies the association between obstructive sleep apnea risk and glaucoma: evidence from the Korea National Health and Nutrition Examination Survey

**DOI:** 10.1186/s40662-025-00436-2

**Published:** 2025-05-13

**Authors:** Jae Hyeok Kwak, Do Young Park, Jong Chul Han

**Affiliations:** 1 Sungkyunkwan University School of Medicine, Seoul, Korea; 2 Department of Ophthalmology, Samsung Medical Center, Sungkyunkwan University School of Medicine, Seoul, Korea; 3 Department of Medical Device, Management and Research, SAIHST, Sungkyunkwan University, Seoul, Korea

**Keywords:** Glaucoma, Sleep duration, Obstructive sleep apnea

## Abstract

**Background:**

This study aimed to investigate the combined effects of obstructive sleep apnea (OSA) risk and short sleep duration on glaucoma prevalence and intraocular pressure (IOP) using data from the 2019 to 2021 Korea National Health and Nutrition Examination Survey (KNHANES).

**Methods:**

This cross-sectional study analyzed data from 7,732 KNHANES participants aged ≥ 40 years. OSA risk was assessed using the STOP-BANG questionnaire, with a high risk defined as a score ≥ 3. The diagnosis of glaucoma was based on the criteria of the International Society of Geographical and Epidemiological Ophthalmology. Multivariate logistic regression models were used to evaluate the associations among glaucoma prevalence, OSA risk, and sleep duration, adjusting for demographic and health-related variables. The interaction effects of OSA risk and sleep duration on glaucoma and IOP were also assessed.

**Results:**

Among the 7,732 participants, 5.28% (n = 408) were diagnosed with glaucoma. Individuals with a high risk of OSA had significantly higher odds of glaucoma compared to those with a low risk (odds ratio: 1.34, 95% confidence interval: 1.02–1.77; *P* < 0.05), with the STOP-BANG components “snoring”, “pressure”, and “age” being most associated with increased glaucoma risk. No significant association was observed between abnormal sleep duration (< 7 h or ≥ 9 h) alone and glaucoma prevalence (*P* > 0.05). Individuals with a high risk of OSA with a sleep duration < 9 h showed a significantly higher glaucoma prevalence than those with ≥ 9 h of sleep (*P* < 0.05), suggesting that sleep duration modifies the association between OSA risk and glaucoma. Similar trends were observed for IOP, with significant interaction effects between OSA risk and sleep duration.

**Conclusions:**

Our findings suggest that sleep duration modulates the association between OSA risk and both glaucoma prevalence and higher IOP, highlighting the importance of including sleep duration in glaucoma risk assessments for patients with OSA. Further research is required to clarify the mechanisms underlying the association between OSA, sleep duration, and glaucoma.

## Background

Glaucoma, a group of progressive optic neuropathies characterized by the degeneration of retinal ganglion cells [1], is the second leading cause of blindness globally, with an estimated prevalence of 4.5% in Korea [2, 3]. Established risk factors include an advanced age, genetic predisposition, myopia, and systemic vascular dysregulation; however, higher intraocular pressure (IOP) remains the most significant factor in glaucoma onset and progression [4]. IOP is the only modifiable risk factor in glaucoma management, and its reduction is currently the principal intervention for decelerating disease progression [5].

Emerging evidence suggests that sleep-related factors, particularly obstructive sleep apnea (OSA), are associated with the risk of glaucoma. OSA, which is characterized by recurrent airway obstruction and intermittent hypoxia, affects 6%–17% of the global population [6]​. This condition is hypothesized to contribute to the pathogenesis of glaucoma via mechanisms such as vascular dysregulation and hypoxic insult to the optic nerve [7]; however, findings on this association remain inconsistent across populations [8–10]. Given the high prevalence of OSA and its potential association with glaucoma, effective screening for OSA is essential. The STOP-BANG questionnaire, a widely used OSA screening tool, has demonstrated acceptable sensitivity (72.4%) and specificity (67.8%) in the Korean population for identifying moderate-to-severe OSA [11].

Sleep duration has also been implicated in glaucoma risk, with one study in the UK population suggesting that both reduced and excessive sleep may increase glaucoma prevalence [12]. In a Korean cross-sectional study, this association was observed primarily in obese individuals [13], underscoring the multifactorial relationship between sleep patterns, comorbid conditions, and glaucoma susceptibility. Recent studies have indicated that sleep duration modulates the effects of OSA on systemic diseases [14, 15]. For instance, patients with OSA with shorter sleep durations exhibit more adverse metabolic outcomes than those with longer sleep durations, suggesting a potential compounding effect between OSA and sleep restriction [16–19]​. However, to the best of our knowledge, no study has examined whether sleep duration influences the association between OSA and the risk of glaucoma.

Using data from the 8th Korea National Health and Nutrition Examination Survey (KNHANES) [20], we investigated whether the combined effect of OSA risk and a short sleep duration significantly increased the risk of glaucoma beyond the independent effects of each factor. Additionally, we assessed associations with IOP, a well-established risk factor for glaucoma, to better understand the role of sleep-related factors in the pathogenesis of glaucoma and the regulation of IOP.

## Methods

### Study design and data source

The KNHANES is a nationwide population-based cross-sectional survey conducted by the Korea Disease Control and Prevention Agency. The survey employs a stratified, multistage cluster sampling design to ensure that the sample is representative of the Korean population. KNHANES collects comprehensive health, nutrition, and lifestyle data through standardized health interviews, physical examinations, and laboratory tests [21].

For this study, we utilized KNHANES data collected from 2019 to 2021. Trained specialists conducted ophthalmic examinations using standardized protocols to assess visual fields, IOP, and optic nerve structure. Visual field tests adhered to reliability criteria, including fixation error ≤ 1 and false-positive rates ≤ 1%. The paper by Song and colleagues describe the methodological details of KNHANES [22].

### Study population

**Fig. 1:**
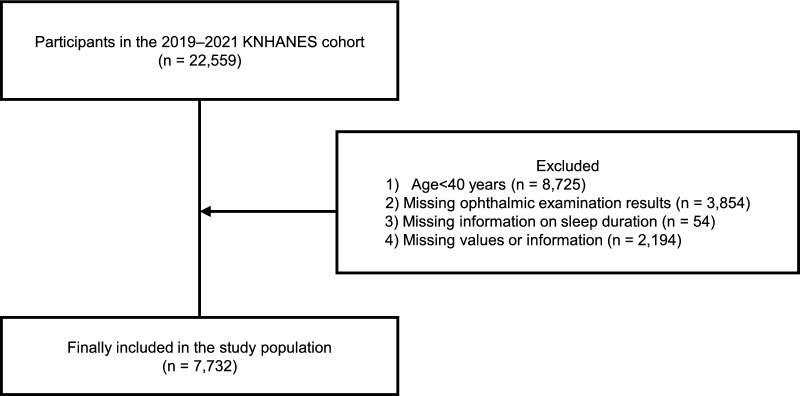
Selection of the study population

Of the 22,559 participants surveyed between 2019 and 2021, 7,732 were included in the final analysis after applying the following exclusion criteria: (1) individuals < 40 years old (n = 8,725), (2) those without ophthalmic examination data (n = 3,854), (3) those lacking sleep duration data (n = 54), and (4) those with missing values for key variables (n = 2,194). The final sample consisted of participants aged ≥ 40 years with complete ophthalmic and sleep-related data, allowing us to investigate the relationship among OSA risk, sleep duration, glaucoma prevalence, and IOP. The process of selecting the study population is summarized in Fig. 1**.**

This study adhered to the guidelines of the Declaration of Helsinki. Institutional Review Board (IRB) approval was obtained from the Samsung Medical Center (IRB No. SMC 2024–08-090; IRB examination exemption approval). The IRB waived the requirement for individual informed consent because of the retrospective design and use of publicly available data. Informed consent was obtained from all participants prior to their enrollment in the KNHANES, in accordance with the ethical guidelines.

### Measurements

Smoking was defined as an adult who had smoked more than five packets of cigarettes (100 cigarettes) during their lifetime. Drinking was defined as drinking alcoholic beverages at least once a month during the past year. Individuals who graduated from high school were classified as having a high level of education. The regions were allocated into two groups: urban (Seoul, Ulsan, Daejeon, Incheon, Busan, Daegu, Gwangju, Sejong, and Gyeonggi) and rural (the rest). Low-income households were categorized as those belonging to the lowest quartile. High physical activity was defined as ≥ 10 metabolic equivalent task (MET)-h/week which is the amount of physical activity recommended by the World Health Organization (WHO) [23]. The MET measures energy expenditure and was calculated using the Global Physical Activity Questionnaire [23]. Stress was defined when responses are “very high” or “high” to the question “What is your stress level in your daily life?”. Body mass index (BMI) was calculated as weight (kg)/height^2^ (m^2^). Waist circumference (cm) was measured at the midlevel between the highest and lowest iliac crests. Neck circumference (cm) was measured in the seated position. Individuals receiving glaucoma treatment were categorized as ‘glaucoma treatment’, whereas others were categorized as ‘no glaucoma treatment’.

Systolic (SBP) and diastolic blood pressure (DBP) were taken as the average values of the second and third trial among three trials. Blood pressure was measured after a minimum of 5 min of rest in a seated position. Hypertension was defined as taking antihypertensive medication, SBP ≥ 140 mmHg, or DBP ≥ 90 mmHg. Total cholesterol, high-density lipoprotein (HDL), fasting plasma glucose (FPG), triglycerides (TG), and glycated hemoglobin (HbA1c) concentrations were measured in blood samples obtained after 8 h of fasting. According to the modified NCEP-ATP III criteria [24], a metabolic syndrome diagnosis was made when an individual met three out of the following five criteria: (1) waist circumference ≥ 90 cm for men and ≥ 80 cm for women, (2) serum TG concentration ≥ 150 mg/dL, (3) serum HDL concentration < 40 mg/dL for men and < 50 mg/dL for women, (4) taking antihypertensive medication or SBP ≥ 130 mmHg or DBP ≥ 85 mmHg, or (5) FPG concentration ≥ 100 mg/dL or undergoing diabetes treatment. Hypertriglyceridemia was defined as TG ≥ 200 mg/dL. Diabetes was defined as a fasting glucose level ≥ 126 mg/dL, HbA1c ≥ 6.5%, current use of oral hypoglycemic agents or insulin injections, or a previous diagnosis of diabetes.

The ophthalmic examinations included visual field tests, IOP measurements, and optical coherence tomography (OCT). Ophthalmic examinations were performed for participants aged 40 years or older in the 2019–2020 survey, and for those who were 19–59 years old in the 2021 survey [22]. The IOP was measured six times, with the final value defined as the average of the six measurements. IOP was measured for both eyes, and the average IOP of both eyes was used for analysis. Refractive error was defined as a mean refractive error of both eyes. Further details of the KNHANES are available in a previously published paper [22].

### Definition of STOP-BANG

The components of STOP-BANG (snoring, tiredness, observed, pressure, BMI, age, neck circumference, gender) were defined as follows [11].

“Snoring”, “tiredness”, and “observed” were defined as a response of “Yes” to the following questions, respectively: “Do you snore loud enough to be heard through closed doors or louder than your voice?”, “Do you often feel tired, fatigued, or sleepful during the daytime?”, and “Did anyone notice if you stopped breathing while you were asleep?”. “Pressure” was defined as an SBP ≥ 140 mmHg or a DBP ≥ 90 mmHg or on antihypertensive medication and “BMI” as those with a BMI > 30 kg/m^2^, modified according to the WHO guidelines for severe obesity in Asians [25]. “Age” was defined as 50 years old or older, “neck” as in neck circumference ≥ 40 cm [11], and “gender” as male.

Participants with a score of 3 or higher on any of the abovementioned eight items were categorized as having a high risk of OSA, and those with a score of 2 or lower as having a low risk of OSA [11].

### Definition of sleep duration

Sleep duration was assessed using a questionnaire. For participants in the 2019–2020 cohort, the question “How long do you usually sleep per day?” was answered in hours (h) for each weekday (working days) and weekend (nonworking days or the day before nonworking days). For participants in the 2021 cohort, the question “When do you usually go to bed and when do you usually wake up?” was answered to the nearest minute on weekdays and weekends. While weekday and weekend sleep durations were obtained from the data, the overall weekday sleep duration was calculated as (5 × weekday sleep duration + 2 × weekend sleep duration)/7.

Abnormal sleep duration was defined as < 7 h or > 9 h, consistent with prior studies linking insufficient and excessive sleep to adverse health outcomes [26, 27]. To examine how sleep duration modifies the relationship between OSA risk and glaucoma or IOP, we performed interaction analyses using specific cutoffs (< 9 h for glaucoma and < 8 h for IOP) identified from observed data patterns.

### Definition of glaucoma

Glaucoma was diagnosed according to the guidelines of the International Society of Geographical and Epidemiological Ophthalmology [28], as the presence of glaucomatous visual field defects with glaucomatous structural changes. Absence of fixation errors and a false-positive error ≤ 1, along with at least one location of reduced sensitivity corresponding to an optic disc or retinal nerve fiber layer abnormality, was defined as a glaucomatous visual field defect. Glaucomatous structural change was defined as the presence of at least one of the following: vertical cup-to-disc ratio ≥ 0.7, neuroretinal rim thinning or notching, a retinal nerve fiber layer defect, or an asymmetrical cup-to-disc ratio ≥ 0.3. When a reliable visual field test result was unavailable, glaucoma was defined by the presence of at least one glaucomatous structural change: thinning or notching of the neuroretinal rim with a cup-to-disc ratio ≥ 0.9, a corresponding retinal nerve fiber layer defect, or an asymmetrical cup-to-disc ratio ≥ 0.3. In the absence of both visual field test results and fundus images, glaucoma was diagnosed by a corrected visual acuity ≤ 10/200 and an IOP exceeding the 97.5th percentile.

### Statistical analysis

All statistical analyses were conducted using R (version 4.3.1; R Foundation for Statistical Computing, Vienna, Austria). The normality of continuous variables was assessed using histograms. Age, refractive error, neck circumference, fasting blood glucose levels, and sleep durations were not normally distributed. Continuous variables are presented as means ± standard error (SE) for consistency across variables and to facilitate interpretation and comparison with previous studies. Categorical variables are presented as proportions (% ± SE).

Multiple logistic regression models for complex survey sampling were applied to examine the association between glaucoma prevalence and the components of the STOP-BANG questionnaire, OSA risk levels, and abnormal sleep duration across different periods (overall week, weekdays, and weekends). These models were adjusted in sequential stages as follows: Model 1 was unadjusted; Model 2 was adjusted for gender and age; Model 3 was additionally adjusted for smoking, drinking, physical activity, education level, and income level; and Model 4 was adjusted for IOP, metabolic syndrome, stress level, and refractive error. For subgroup analysis, the association between OSA risk and glaucoma prevalence was examined within each sleep duration category (< 6 h, 6 h ~ < 7 h, 7 h ~ < 8 h, 8 h ~ < 9 h, and ≥ 9 h) for overall week, weekday, and weekend durations. Interaction terms were included to assess the effect of sleep duration modification (< 9 h vs. ≥ 9 h) on the relationship between OSA risk and glaucoma prevalence.

The relationship between IOP and the components of the STOP-BANG questionnaire, OSA risk, and abnormal sleep duration across the same time periods were assessed using multiple linear regression for complex sampling. Associations between OSA risk and IOP were further evaluated within each sleep duration subgroup (< 6 h, 6 h ~ < 7 h, 7 h ~ < 8 h, 8 h ~ < 9 h, and ≥ 9 h) across the overall week, weekday, and weekend periods using Student’s *t-*test, and interaction terms were included in the linear regression models to investigate the interaction between sleep duration (< 8 h vs*.* ≥ 8 h) and OSA risk for effects on IOP. Adjustments were applied in the same sequence across the models as described above, except for Model 4, which excluded the IOP to avoid repetition and included glaucoma treatment. In models that analyzed individual components of the STOP-BANG questionnaire, age and gender variables were excluded for the respective STOP-BANG components “age” (≥ 50 years) and “gender” (male) to avoid redundancy.

All statistical tests were two-sided, with a *P* value of less than 0.05 indicating statistical significance.

## Results

The clinical characteristics of participants with and without glaucoma are summarized in Table 1. Among the 7,732 participants, 408 (5.28%) were diagnosed with glaucoma, which is similar to the previously reported prevalence of approximately 4.5% in the Korean population aged ≥ 40 years [2, 3]. The mean age, waist circumference, neck circumference, systolic BP, and fasting glucose levels were significantly higher in participants with glaucoma than in those without glaucoma (all *P* < 0.05). Moreover, participants with glaucoma were more likely to be myopic than those without glaucoma (*P* < 0.05). In contrast, those without glaucoma had higher mean levels of HDL cholesterol, a higher proportion of individuals with a high educational level and reported stress (all *P* < 0.05). Furthermore, participants with glaucoma exhibited a higher prevalence of hypertension, hypercholesterolemia, diabetes, smoking, a low income, metabolic syndrome, and glaucoma treatment than those without glaucoma (all *P* < 0.05).

**Table 1:** Characteristics of the study population

Variable	Glaucoma: No (n = 7324)	Glaucoma: Yes (n = 408)	*P* value
Age (years)	55.00 (0.25)	59.86 (0.81)	< 0.001
IOP (mmHg)	14.67 (0.05)	14.95 (0.17)	0.083
Hypertension (Yes, %)	33.79 (0.01)	51.31 (0.03)	< 0.001
Hypercholesterolemia (Yes, %)	30.41 (0.01)	37.68 (0.03)	0.013
Hypertriglyceridemia (Yes, %)	15.94 (0.01)	16.65 (0.02)	0.749
Diabetes (Yes, %)	15.72 (0.01)	26.18 (0.03)	< 0.001
Waist circumference (cm)	85.02 (0.15)	87.72 (0.53)	< 0.001
BMI (kg/m^2^)	24.28 (0.05)	24.62 (0.19)	0.080
Neck circumference (cm)	35.31 (0.05)	36.17 (0.20)	< 0.001
Systolic BP (mmHg)	120.32 (0.28)	124.17 (0.99)	< 0.001
Diastolic BP (mmHg)	76.95 (0.16)	76.66 (0.58)	0.646
Refractive error (D)	− 0.51 (0.04)	− 1.09 (0.20)	0.003
Stress (%)	24.30 (0.01)	17.78 (0.02)	0.011
Fasting glucose (mg/dL)	103.18 (0.34)	107.23 (1.67)	0.018
HDL (mg/dL)	51.95 (0.2)	49.25 (0.68)	< 0.001
Smoking (Yes, %)	40.96 (0.01)	48.21 (0.03)	0.016
Drinking (Heavy, %)	52.25 (0.01)	53.07 (0.03)	0.798
Education level (High, %)	77.20 (0.01)	66.65 (0.03)	< 0.001
Residence area (Urban, %)	70.61 (0.02)	70.55 (0.03)	0.986
Income (Low, %)	14.39 (0.01)	20.15 (0.02)	0.002
MetS (Yes, %)	35.87 (0.01)	45.85 (0.03)	< 0.001
Physical activity (Yes, %)	41.76 (0.01)	38.54 (0.03)	0.295
Overall week sleep duration (hour)	6.80 (0.02)	6.66 (0.08)	0.086
Weekday sleep duration (hour)	6.64 (0.02)	6.52 (0.08)	0.144
Weekend sleep duration (hour)	7.19 (0.02)	7.00 (0.10)	0.070
Glaucoma treatment (%)	13.33 (0.02)	0.39 (0.00)	< 0.001

*IOP* = intraocular pressure; *BMI* = body mass index; *BP* = blood pressure; *HDL* = high-density lipoprotein; *MetS* = metabolic syndrome

Columns “Glaucoma: Yes” and “Glaucoma: No” indicate the presence and absence of glaucoma, respectively

Data are presented as the mean standard error (SE) or % (SE)

### OSA risk (STOP-BANG questionnaire) and glaucoma prevalence

The association between STOP-BANG components and glaucoma prevalence was evaluated across the four models (Table 2). “Snoring,” “pressure,” and “age,” were the STOP-BANG components consistently associated with a higher likelihood of glaucoma in all the models, and “gender” was associated with a higher glaucoma prevalence in models 1–3. For instance, in Model 4, “snoring” was independently associated with glaucoma [adjusted odds ratio (aOR): 1.43; 95% CI: 1.07–1.91; *P* = 0.015]. In the fully adjusted Model 4, individuals with a high risk of OSA (STOP-BANG score ≥ 3) had significantly higher odds of glaucoma than those with a low risk (STOP-BANG score < 3) (OR: 1.34, 95% CI: 1.02–1.77; *P* = 0.034). These results highlight a robust and independent association between OSA risk and glaucoma prevalence, even after adjusting for confounding variables, such as age, gender, and IOP.

**Table 2:** Relationship between OSA risk (STOP-BANG) and the prevalence of glaucoma

	Unadjusted		Model 2		Model 3		Model 4	
STOP-BANG components	OR (95% Cl)	*P* value	aOR (95% Cl)	*P* value	aOR (95% Cl)	*P* value	aOR (95% Cl)	*P* value
Snoring	1.50 (1.14–1.97)	0.004	1.48 (1.12–1.96)	0.006	1.48 (1.12–1.96)	0.007	1.43 (1.07–1.91)	0.015
Tired	0.96 (0.72–1.27)	0.756	1.02 (0.76–1.36)	0.897	1.02 (0.76–1.36)	0.919	1.03 (0.77–1.39)	0.825
Observed apnea	1.35 (0.91–2.00)	0.140	1.28 (0.87–1.88)	0.210	1.27 (0.87–1.86)	0.220	1.20 (0.81–1.78)	0.354
Pressure	2.06 (1.60–2.66)	< 0.001	1.56 (1.20–2.04)	0.001	1.55 (1.18–2.04)	0.002	1.45 (1.09–1.93)	0.011
BMI	1.08 (0.66–1.77)	0.750	1.26 (0.77–2.09)	0.360	1.26 (0.76–2.09)	0.367	1.12 (0.66–1.88)	0.676
Age	1.76 (1.24–2.50)	0.002	1.79 (1.26–2.53)	0.001	1.53 (1.06–2.21)	0.025	1.80 (1.21–2.68)	0.004
Neck	1.42 (0.97–2.07)	0.071	1.41 (0.94–2.11)	0.095	1.41 (0.94–2.10)	0.099	1.19 (0.79–1.78)	0.399
Gender	1.48 (1.17–1.87)	0.001	1.56 (1.24–1.97)	< 0.001	1.45 (1.01–2.10)	0.046	1.38 (0.96–1.99)	0.084
OSA high risk group (STOP-BANG score ≥ 3)	1.87 (1.49–2.35)	< 0.001	1.40 (1.07–1.84)	0.014	1.39 (1.06–1.82)	0.017	1.34 (1.02–1.77)	0.034

*OR* = odds ratio; *aOR* = adjusted odds ratio; *CI* = confidence interval; *BMI* = body mass index; *OSA* = obstructive sleep apnea

ORs and 95% CIs were calculated using logistic regression models. Adjustments were made progressively in Models 2–4 for demographic and health-related variables as specified

Model 2: Adjusted for gender and age

Model 3: Adjusted for variables in Model 2, with additional adjustments for smoking, drinking, physical activity, educational level, and income level

Model 4: Adjusted for variables in Model 3, with additional adjustments for intraocular pressure, metabolic syndrome, stress level, and refractive error

Age and gender variables were excluded for the respective STOP-BANG components “age” (≥ 50 years) and “gender” (male) to avoid redundancy

### Sleep duration and glaucoma prevalence

**Fig. 2:**
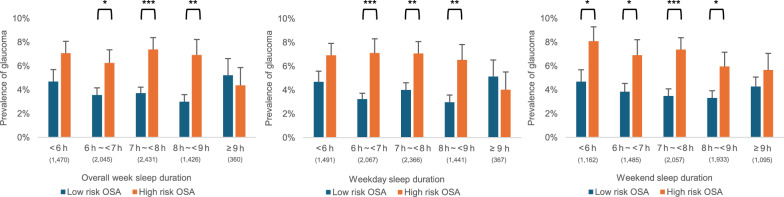
Relationship between the risk of obstructive sleep apnea (OSA) and prevalence of glaucoma in each sleep duration subgroup. The number of participants (n) in each subgroup is shown in parentheses. *, **, and *** indicate *P* values of < 0.05, < 0.01, and < 0.001, respectively

Overall analysis showed no direct association between sleep duration and glaucoma prevalence (*P* > 0.05; Table 3). However, subgroup analysis revealed that sleeping for ≥ 9 h made a marked shift in the association between OSA risk and glaucoma prevalence (Fig. 2). Specifically, the association between OSA risk and glaucoma prevalence was more pronounced for sleep durations < 9 h, whereas it became statistically non-significant for durations ≥ 9 h. These findings suggest that the effects of OSA risk on glaucoma prevalence are modulated by sleep duration.

**Table 3:** Relationship between sleep duration and the prevalence of glaucoma

	Unadjusted		Model 2		Model 3		Model 4	
Abnormal sleep (< 7 h or ≥ 9 h)	OR (95% Cl)	*P* value	aOR (95% Cl)	*P* value	aOR (95% Cl)	*P* value	aOR (95% Cl)	*P* value
Overall week	1.04 (0.81–1.33)	0.750	0.97 (0.76–1.24)	0.813	0.97 (0.76–1.24)	0.819	0.98 (0.77–1.26)	0.896
Weekday	1.05 (0.83–1.32)	0.683	0.99 (0.78–1.24)	0.903	0.99 (0.78–1.25)	0.909	0.99 (0.78–1.25)	0.918
Weekend	1.16 (0.91–1.48)	0.242	1.09 (0.85–1.39)	0.502	1.09 (0.85–1.39)	0.505	1.11 (0.87–1.42)	0.416

*OR* = odds ratio; *aOR* = adjusted odds ratio; *CI* = confidence interval

Logistic regression was used to estimate ORs and 95% CIs. Adjustments followed the same model progression as that in Table 2

Model 2: Adjusted for gender and age

Model 3: Adjusted for variables in Model 2, with additional adjustments for smoking, drinking, physical activity, educational level, and income level

Model 4: Adjusted for variables in Model 3, with additional adjustments for intraocular pressure, metabolic syndrome, stress level, and refractive error

### Interaction between sleep duration and OSA risk on glaucoma prevalence

Table 4 presents the interaction between sleep duration (< 9 h vs. ≥ 9 h), OSA risk, and the prevalence of glaucoma. A sleep duration threshold of < 9 h was determined based on the observed data patterns, where the association between OSA risk and glaucoma prevalence exhibited significant transitions (Fig. 2). Although no significant interaction was observed in the unadjusted model, significant interactions emerged in Models 2, 3, and 4 for the overall week and weekday sleep durations. In Model 4, sleeping for < 9 h significantly increased the effect of OSA risk on glaucoma prevalence, with aORs of 2.72 (95% CI: 1.07–6.92; *P* = 0.035) for the overall week sleep duration and 2.93 (95% CI: 1.11–7.74; *P* = 0.031) for weekday sleep duration.

**Table 4:** Effects of the interaction between a sleep duration (< 9 h) and OSA risk on glaucoma prevalence

Sleep duration (< 9 h)	Unadjusted		Model 2		Model 3		Model 4	
	OR (95% Cl)	*P* value	aOR (95% Cl)	*P* value	aOR (95% Cl)	*P* value	aOR (95% Cl)	*P* value
Overall week	2.34 (0.92–5.94)	0.074	2.78 (1.10–7.00)	0.031	2.80 (1.11–7.07)	0.030	2.72 (1.07–6.92)	0.035
Weekday	2.50 (0.95–6.61)	0.064	2.94 (1.11–7.68)	0.030	2.94 (1.12–7.73)	0.029	2.93 (1.11–7.74)	0.031
Weekend	1.47 (0.74–2.93)	0.275	1.58 (0.79–3.18)	0.194	1.59 (0.80–3.20)	0.187	1.76 (0.88–3.52)	0.111

*OR* = odds ratio; *aOR* = adjusted odds ratio; *CI *= confidence interval; *OSA* = obstructive sleep apnea

Logistic regression models included interaction terms for sleep duration and OSA risk. Adjustments were applied as outlined in the model specifications

Model 2: Adjusted for gender and age

Model 3: Adjusted for variables in Model 2, with additional adjustments for smoking, drinking, physical activity, educational level, and income level

Model 4: Adjusted for variables in Model 3, with additional adjustments for intraocular pressure, metabolic syndrome, stress level, and refractive error

### OSA risk (STOP-BANG questionnaire) and IOP

The relationship between STOP-BANG components and IOP was assessed across all models (Table 5). The STOP-BANG parameters “snoring,” “pressure,” “BMI,” and “neck” were significantly associated with higher IOP, while “age” was consistently associated with lower IOP in all models. In Model 4, “snoring” was independently associated with higher IOP (β = 0.24; 95% CI: 0.10–0.38; *P* < 0.001). A high risk of OSA (STOP-BANG score ≥ 3) was significantly associated with higher IOP in Model 4 (β = 0.34; 95% CI: 0.20–0.47; *P* < 0.001). These findings indicate a strong and independent relationship between OSA risk and IOP.

**Table 5:** Relationship between OSA risk (STOP-BANG) and intraocular pressure

STOP-BANG components	Unadjusted			Model 2			Model 3			Model 4		
	β	95% CI	*P* value	β	95% CI	*P* value	β	95% CI	*P* value	β	95% CI	*P* value
Snoring	0.39	0.26–0.53	< 0.001	0.34	0.20–0.47	0.131	0.32	0.19–0.46	< 0.001	0.24	0.10–0.38	< 0.001
Tired	0.06	− 0.07–0.18	0.381	0.03	− 0.09–0.15	0.605	0.03	− 0.09–0.15	0.627	0.02	− 0.10–0.15	0.701
Observed	0.32	0.11–0.52	0.003	0.22	0.01–0.43	0.038	0.21	0.00–0.42	0.055	0.13	− 0.07–0.33	0.213
Pressure	0.18	0.05–0.31	0.008	0.47	0.34–0.61	< 0.001	0.48	0.34–0.62	< 0.001	0.30	0.16–0.44	< 0.001
BMI	0.67	0.41–0.93	< 0.001	0.54	0.29–0.79	< 0.001	0.57	0.32–0.82	< 0.001	0.32	0.07–0.58	0.013
Age	− 0.46	− 0.60 to − 0.32	< 0.001	− 0.46	− 0.60 to − 0.32	< 0.001	− 0.30	− 0.44 to − 0.15	< 0.001	− 0.21	− 0.36 to − 0.07	0.004
Neck	0.82	0.61–1.03	< 0.001	0.70	0.48–0.92	< 0.001	0.70	0.47–0.92	< 0.001	0.49	0.27–0.71	< 0.001
Gender	0.17	0.06–0.28	0.002	0.14	0.03–0.25	0.013	− 0.07	− 0.23–0.10	0.437	− 0.13	− 0.30–0.04	0.129
OSA high risk group (STOP-BANG score ≥ 3)	0.26	0.14–0.38	< 0.001	0.47	0.33–0.61	< 0.001	0.46	0.32–0.60	< 0.001	0.34	0.20–0.47	< 0.001

*CI* = confidence interval; *BMI* = body mass index

Linear regression models were used to estimate β coefficients and 95% CIs for intraocular pressure. Adjustments were progressively included in Models 2–4

Model 2: Adjusted for gender and age

Model 3: Adjusted for variables in Model 2, with additional adjustments for smoking, drinking, physical activity, educational level, and income level

Model 4: Adjusted for variables in Model 3, with additional adjustments for metabolic syndrome, stress level, refractive error, and glaucoma treatment

Age and gender variables were excluded for the respective STOP-BANG components “age” (≥ 50 years) and “gender” (male) to avoid redundancy

### Sleep duration and IOP

**Fig. 3:**
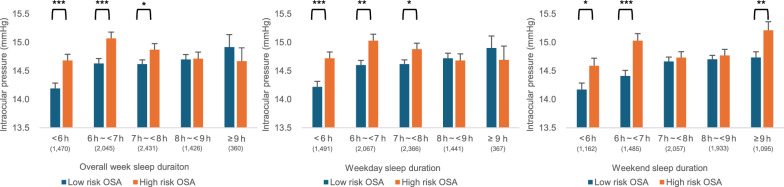
Relationship between the risk of obstructive sleep apnea (OSA) and intraocular pressure (IOP) in each sleep duration subgroup. The number of participants (n) in each subgroup is shown in parentheses. *, **, and *** indicate *P* values of < 0.05, < 0.01, and < 0.001, respectively

Overall analysis showed no significant association between sleep duration and IOP (Table 6). However, subgroup analysis revealed that sleeping for ≥ 8 h made a marked shift in the association between OSA risk and IOP (Fig. 3). Specifically, the association was significant for sleep durations < 8 h, while it became statistically non-significant for durations ≥ 8 h for the overall week and weekday sleep durations.

**Table 6:** Relationship between sleep duration and intraocular pressure

Abnormal sleep (< 7 h or ≥ 9 h)	Unadjusted			Model 2			Model 3			Model 4		
	β	95% CI	*P* value	β	95% CI	*P* value	β	95% CI	*P* value	β	95% CI	*P* value
Overall week	− 0.06	− 0.18–0.07	0.364	0.01	− 0.12–0.13	0.933	0.00	− 0.12–0.12	0.963	− 0.01	− 0.13–0.11	0.865
Weekday	− 0.06	− 0.19–0.06	0.315	− 0.01	− 0.13–0.11	0.854	− 0.01	− 0.13–0.11	0.828	− 0.03	− 0.15–0.09	0.621
Weekend	− 0.05	− 0.17–0.06	0.362	0.01	− 0.10–0.12	0.899	0.01	− 0.11–0.12	0.913	0.00	− 0.11–0.11	0.969

*CI* = confidence interval

Linear regression was applied to calculate β coefficients and 95% CIs. Adjustments were progressively included in Models 2–4

Model 2: Adjusted for gender and age

Model 3: Adjusted for variables in Model 2, with additional adjustments for smoking, drinking, physical activity, educational level, and income level

Model 4: Adjusted for variables in Model 3, with additional adjustments for metabolic syndrome, stress level, refractive error, and glaucoma treatment

### Interaction between sleep duration and OSA risk on IOP

Table 7 shows the interaction between sleep duration (< 8 h vs. ≥ 8 h), OSA risk, and IOP. A sleep duration threshold of < 8 h, derived from data patterns showing a marked shift in the relationship between OSA risk and IOP, was used to analyze their interaction. A significant interaction between OSA risk and an overall week/weekday sleep duration < 8 h was observed across all models (Model 4: overall week sleep duration β = 0.29; 95% CI: 0.02–0.55; *P* = 0.035; weekday sleep duration β = 0.34; 95% CI: 0.07–0.60; *P* = 0.013). These findings indicate that a sleep duration < 8 h amplified the effect of OSA risk on IOP.

**Table 7:** Effects of the interaction between sleep duration (< 8 h) and OSA risk on intraocular pressure

	Unadjusted			Model 2			Model 3			Model 4		
Sleep duration (< 8 h)	β	95% CI	*P* value	β	95% CI	*P* value	β	95% CI	*P* value	β	95% CI	*P* value
Overall week	0.39	0.11–0.66	0.006	0.29	0.02–0.56	0.038	0.29	0.02–0.56	0.038	0.29	0.02–0.55	0.035
Weekday	0.44	0.16–0.71	0.002	0.34	0.07–0.61	0.013	0.34	0.07–0.61	0.013	0.34	0.07–0.60	0.013
Weekend	0.10	− 0.15–0.35	0.433	0.02	− 0.22–0.27	0.842	0.02	− 0.23–0.26	0.895	0.05	− 0.19–0.29	0.664

*CI* = confidence interval; *BMI* = body mass index; *OSA* = obstructive sleep apnea

Linear regression models included interaction terms for sleep duration and OSA risk. Adjustments for covariates were made progressively in Models 2–4

Model 2: Adjusted for gender and age

Model 3: Adjusted for variables in Model 2, with additional adjustments for smoking, drinking, physical activity, educational level, and income level

Model 4: Adjusted for variables in Model 3, with additional adjustments for metabolic syndrome, stress level, refractive error, and glaucoma treatment

## Discussion

This study investigated the association among OSA risk, sleep duration, and glaucoma using data from the KNHANES. Using the STOP-BANG questionnaire to assess OSA risk, we found a significant association among OSA risk, glaucoma prevalence, and higher IOP. Notably, although sleep duration alone was not associated with glaucoma or IOP, our results demonstrated a significant interaction between sleep duration and OSA risk for their impact on glaucoma risk, indicating that a shorter sleep duration may exacerbate the ocular effects of OSA, further elucidating the complexity of the relationship between these conditions.

OSA has long been recognized as a potential risk factor for glaucoma, with previous studies presenting inconsistent findings regarding the magnitude and nature of this association. Some studies have reported a significant association between OSA and glaucoma [29, 30], whereas others have not [9, 31, 32]. These discrepancies may stem from differences in the study populations and methodologies and the omission of key factors, such as sleep duration. Our findings suggested that sleep duration plays an important role in modulating the relationship between OSA risk and glaucoma, which has not been adequately addressed in previous studies. In this study, we accounted for sleep duration by stratifying the participants accordingly, which helped clarify inconsistent findings and provided a more detailed understanding on how OSA contributes to the risk of glaucoma.

Mechanistically, several pathways may explain the interactions between OSA, short sleep duration, and glaucoma. Prior studies have shown that shorter sleep duration is more frequently associated with severe OSA than with mild or moderate OSA [14]. In patients with OSA, short sleep duration has been linked to elevated levels of inflammatory markers, such as myeloperoxidase and oxidized low-density lipoprotein, which exacerbate oxidative stress and contribute to retinal ganglion cell damage and optic nerve degeneration [33]. These findings highlight how short sleep in patients with OSA amplifies systemic inflammation and vascular dysregulation, which are key mechanisms in glaucoma pathogenesis [34]. While longer sleep durations could theoretically increase the cumulative hypoxic burden due to prolonged intermittent hypoxia, previous studies have shown that longer sleep duration in patients with OSA is significantly associated with higher mean oxygen saturation and a lower risk of hypertension [35, 36], even after adjusting for the apnea–hypopnea index (AHI). These findings suggest that extended sleep may mitigate some of the cardiovascular and hypoxic effects of OSA. In contrast, shorter sleep duration imposes distinct physiological stressors, including greater sleep fragmentation, characterized by increased arousal index values and prolonged wake after sleep onset [15]. These disruptions are closely linked to heightened sympathetic nervous system activity and vascular instability [37], compounding the deleterious ocular effects of OSA. Importantly, the interaction between short sleep duration and OSA risk indicates that short sleep is not merely a proxy for OSA severity, but also an independent modifier of glaucoma risk. By intensifying vascular and hypoxic stress on the optic nerve, short sleep duration probably exacerbates the adverse effects of OSA. Further research incorporating direct metric of OSA severity, AHI, is essential to clarify the interplay between sleep duration, OSA severity, and glaucoma.

Our study observed a minimal difference in mean IOP between the glaucoma and control groups, both of which had values comparable to the average of approximately 14.10 mmHg in the Korean population [38]. This likely reflects the high prevalence of normal tension glaucoma (NTG) in Korea, where approximately 60% of cases occur with IOP within the normal range [39]. In our study, a significant proportion of patients with glaucoma are likely to have NTG. Shared mechanisms, including vascular dysregulation, intermittent hypoxia, and oxidative stress, may underlie the association between OSA and NTG, independent of IOP [40, 41]. Additionally, IOP-lowering treatments in many patients with glaucoma may have further attenuated the observed differences. These findings highlight the need to investigate the non-IOP-mediated pathways linking OSA and glaucoma, particularly in populations with a high prevalence of NTG.

In clinical practice, the STOP-BANG questionnaire is commonly used to identify individuals at high risk of OSA (STOP-BANG score ≥ 3) [42]. The STOP-BANG score was analyzed with a categorical cutoff in our study, which complements a previous study that applied a discrete variable [43] and facilitates clinical interpretation. This approach aligns with standard screening protocols and ensures consistency with previous studies, enabling its practical application in clinical settings. Additionally, our analysis of the individual STOP-BANG components identified “snoring” as an independent risk factor for glaucoma, corroborating previous findings from a UK Biobank study that linked snoring with an increased risk of glaucoma [12]. An association between snoring and higher IOP was observed in this study. These findings further support the hypothesis that snoring, a common symptom of OSA, contributes to glaucomatous damage through mechanisms related to intermittent hypoxia and the inflammatory response [44].

Contrary to earlier studies (using UK Biobank data) that reported associations between abnormal sleep duration (both short and long sleep periods) and glaucoma [12], our findings did not reveal a significant relationship between sleep duration and glaucoma in the general population. This result is consistent with that of a previous study using the KNHANES data, which found an association between sleep duration and glaucoma only in overweight individuals [13]. These discrepancies between studies may reflect differences in genetic, environmental, and methodological factors. The higher prevalence of NTG in East Asians may partially explain the population-specific variation [3]. Additionally, regional differences in lifestyle, healthcare access, and comorbidities such as obesity or hypertension can modulate the relationship between sleep duration and glaucoma [45, 46]. Methodological variations, including differences in sampling strategies and definitions of abnormal sleep duration, may have further contributed to these discrepancies. Furthermore, while no direct association was found between sleep duration and IOP in the overall population, subgroup analyses suggested that this relationship may vary with OSA risk. This lack of a clear association underscores the complexity of the interactions between sleep duration, OSA, and glaucoma, highlighting the need for further investigations into potential non-uniform relationships and subgroup-specific effects.

Although our study had strengths such as the use of a large nationally representative dataset and well-validated OSA screening tool, several limitations should be noted. First, sleep duration was self-reported, which may have introduced a recall bias and reduced the accuracy of our sleep assessments. Objective measures of sleep such as polysomnography and actigraphy can provide more precise estimates of sleep duration and its impact on glaucoma. Second, the STOP-BANG questionnaire, although validated and practical for large-scale studies, does not provide a definitive diagnosis of OSA. This may have influenced the accuracy of the OSA risk classification in our study. Moreover, the questionnaire may not capture the full spectrum of OSA severity, particularly nocturnal hypoxia, which is hypothesized to play a direct role in glaucoma pathogenesis [47]. Finally, the study population included only Koreans, which restricts the generalizability of the results to other populations. Future studies involving diverse populations are required to validate these findings.

## Conclusions

Our study provides evidence for a significant interaction between a sleep duration and OSA risk in relation to glaucoma prevalence and IOP, suggesting that sleep duration is a crucial factor that should be considered when assessing glaucoma risk associated with OSA. These findings emphasize the importance of addressing both sleep duration and OSA in the management of patients at risk of glaucoma. Further research is warranted to explore the potential benefits of sleep interventions in mitigating the progression of glaucoma and to better understand the underlying mechanisms linking OSA, sleep duration, IOP, and glaucoma.

## Data Availability

The datasets analyzed during the current study are available in the KNHANES repository, https://knhanes.kdca.go.kr/knhanes/sub03/sub03_02_05.do.

## Abbreviations

KNHANES: Korea National Health and Nutrition Examination Survey
IOP: Intraocular pressure
OSA: Obstructive sleep apnea
IRB: Institutional Review Board
MET: Metabolic equivalent task
WHO: World Health Organization
BMI: Body mass index
SBP: Systolic blood pressure
DBP: Diastolic blood pressure
HDL: High-density lipoprotein
FPG: Fasting plasma glucose
TG: Triglycerides
OCT: Optical coherence tomography
OR: Odds ratio
aOR: Adjusted odds ratio
CI: Confidence interval
